# Barriers and facilitators of adherence to TB treatment in patients on concomitant TB and HIV treatment: a qualitative study

**DOI:** 10.1186/1471-2458-10-651

**Published:** 2010-10-28

**Authors:** Mekdes K Gebremariam, Gunnar A Bjune, Jan C Frich

**Affiliations:** 1 Section for International Health, Institute of Health and Society, University of Oslo, PO Box 1130, Blindern, NO-0318 Oslo, Norway; 2 Department of Health Management and Health Economics, Institute of Health and Society, University of Oslo, PO Box 1089, NO-0317 Oslo, Norway

## Abstract

**Background:**

Tuberculosis is a major public health problem in Ethiopia, and a high number of TB patients are co-infected with HIV. There is a need for more knowledge about factors influencing treatment adherence in co-infected patients on concomitant treatment. The aim of the present study is to explore patients' and health care professionals' views about barriers and facilitators to TB treatment adherence in TB/HIV co-infected patients on concomitant treatment for TB and HIV.

**Methods:**

Qualitative study using in-depth interviews with 15 TB/HIV co-infected patients and 9 health professionals and focus group discussions with 14 co-infected patients.

**Results:**

We found that interplay of factors is involved in the decision making about medication intake. Factors that influenced adherence to TB treatment positively were beliefs in the curability of TB, beliefs in the severity of TB in the presence of HIV infection and support from families and health professionals. Barriers to treatment adherence were experiencing side effects, pill burden, economic constraints, lack of food, stigma with lack of disclosure, and lack of adequate communication with health professionals.

**Conclusion:**

Health professionals and policy makers should be aware of factors influencing TB treatment in TB/HIV co-infected patients on concomitant treatment for TB and HIV. Our results suggest that provision of food and minimal financial support might facilitate adherence. Counseling might also facilitate adherence, in particular for those who start ART in the early phases of TB treatment, and beliefs related to side-effects and pill burden should be addressed. Information to the public may reduce TB and HIV related stigma.

## Background

Tuberculosis is a major public health problem in Ethiopia, with an estimated incidence of 379 per 100 000 and a prevalence of 643 per 100 000 [1]. According to hospital statistics data, TB is the leading cause of morbidity, the third cause of hospital admission and the second cause of death in Ethiopia [2]. There is a high rate of HIV among patients with TB in Ethiopia. The WHO Global Report 2008 estimated that 40% of patients treated for TB are HIV positive [2].

Effective treatment exists for both TB and HIV. Evidence shows that ART treatment can have a significant impact on the HIV and TB related morbidity and mortality in co-infected patients; but concomitant treatment is complicated by factors such as overlapping drug toxicities, drug-drug interactions and possible paradoxical reactions [3-7]. Concomitant treatment also leads to a higher pill burden. There is an association between poor adherence and the pill burden associated with a regimen, the regimen's complexity, and the extent to which the regimen impacts on the patient's daily life [8-11]. Co-infected patients on concomitant treatment may thus be at risk for decreased adherence to either or both treatments. Low adherence to TB treatment can lead to an increased risk of drug resistance, relapse, death, and may prolong infectiousness [12].

Adherence is defined by WHO as "the extent to which a person's behavior- taking medication, following a diet, and or executing lifestyle changes, corresponds with agreed recommendations from a health care provider" [13]. Adherence it is a concept that allows for a comprehensive assessment of factors related to medication intake such as characteristics of the regimen; attitudes of the providers; socio-economic, cultural and environmental factors [14,15].

Studies of patients' adherence to TB treatment in Ethiopia have identified socio-economic and structural barriers to adherence, including lack of money to pay for transportation, high distance from clinics, the difficulties associated with daily treatment and rigid routines at health facilities [12,16-23]. Inadequate knowledge about the illness was also found to adversely impact adherence. The studies also showed that family support, involvement in TB clubs, and adequate knowledge about the disease were facilitators of TB treatment adherence [12,16-23].

To our knowledge, no study has explored the experiences of TB/HIV co-infected patients receiving concomitant treatment in Ethiopia. How concomitant treatment affects TB treatment adherence has similarly not been explored. A review of qualitative studies of factors considered as important by patients, caregivers and health care providers for TB medication adherence indicated a lack of evidence on the experiences of patients living with HIV/AIDS and taking treatment for TB or for both illnesses [11]. There is a need for more knowledge about factors influencing treatment adherence in co-infected patients on concomitant treatment. We thus conducted a study to explore patients' and health care professionals' views about barriers and facilitators to TB treatment adherence in co-infected patients who received concomitant treatment for TB and HIV.

## Methods

### Setting

The study was conducted in Addis-Ababa, the capital city of Ethiopia, in the period July 2008 to October 2008. The health service coverage in Addis-Ababa, with regards to geographical accessibility, is 100% [24]. Out-patient TB treatment is mainly provided by governmental health centers. These health centers are administered by the Addis Ababa Health Bureau and provide medical services for the populations within their catchments areas. TB clinics have been operating for many years in local health centers, and ART clinics were established in 2006, as part of the expansion of HAART provision program initially started in hospitals [25]. The health centers offer free ART service and care for patients with HIV infection. In addition to diagnosis and treatment of patients with TB, the TB clinics also offer the Provider Induced Counseling and Treatment (PICT) service through which all patients coming to the TB clinics are offered screening for HIV. Both TB and ART treatments are offered free of charge in these health centers. The different health centers in general do not have significant differences in terms of services they provide and in terms of health personnel and equipments available.

### Participants

We collected data using in-depth interviews and focus group discussions. We conducted individual interviews with 15 patients (7 men, 8 women) who had been on concomitant treatment for TB and HIV, and we interviewed nine health professionals (6 clinical nurses, 2 health officers and 1 doctor) who were involved in the treatment of these patients. We conducted a preliminary analysis of our in-depth interviews, identifying major themes emerging from our data. We subsequently conducted two focus group discussions with a total of 14 patients (7 men, 7 women), in order to further explore and validate our preliminary findings from the in-depth interviews. Participants were recruited from three out of 21 health centres: Bole Health Center, Arada Health Center and Woreda 7 Health Center.

We used a maximum variation sampling strategy when recruiting participants, to ensure diversity in views and experiences. We thus included participants with diversity with regards to age, gender, education, and occupation. In addition, we included patients for whom ART was started at different stages during TB treatment (before anti-TB initiation, in intensive phase, in continuation phase). We ensured diversity of treatment experiences by interviewing an equal number of patients who had defaulted treatment and with successful treatment; we also included three patients who were on retreatment.

Patients with pre-specified characteristics were invited to participate in the study through an invitation letter provided by health personnel. Patients who had defaulted treatment were contacted by health personnel on our behalf while being traced after treatment discontinuation at the TB clinics and two were contacted through personnel at the ART clinic. Only one patient declined to participate in the study. Health personnel were directly invited by the first author (MK) to participate in the study. We interviewed patients who had either completed or discontinued treatment in the past six months before data collection.

### Data collection

Audio-recorded individual interviews and focus group discussions were conducted by MK in Amharic. Individual interviews took place in private rooms, and a semi-structured format with open-ended questions was used. The questions covered participants' perceptions about TB, HIV and co-infection; experiences of concomitant medication intake; factors positively and adversely influencing TB medication intake while on concomitant ART treatment; and experiences at both TB and HIV clinics. Similar topics were covered in the focus group discussions, and issues that arose in the interviews were brought up for further discussion. We used this triangulation of data sources in order to increase the validity of findings in our study.

We developed a semi-structured interview guide for patients that was inspired by a theoretical framework for health related behaviors, in which the following six major categories are essential: accessibility of health care; evaluation of health care; perception of symptoms and threat of disease; social network characteristics; knowledge about disease; demographic characteristics [26]. The interview guide was also inspired by further review of literature on adherence which indicates the importance of other factors such as regimen related factors and health service related factors [8-11,27].

We developed an interview guide for health workers, and we explored their experiences with adherence to treatment in co-infected patients on concomitant treatment, with a focus on barriers and facilitators to adherence. Other questions such as what they do to try and assist patients with adherence, what they would recommend would assist patients with their medication intake were also included.

### Data analysis

Audio-recorded data from interviews of patients was transcribed verbatim and translated into English. A preliminary analysis of interviews was done, and used for validation of results and further exploration using focus group interviews. Data from health worker interviews and focus group discussions which were also audio-recorded were also transcribed verbatim and translated into English.

The final analysis involved identification and coding of material about participants' perceptions about TB and HIV, perceived barriers and facilitators of TB treatment adherence using Giorgi's phenomenological method, as modified by Malterud [28]. MK read the material to get an overview and subsequently identified units of meaning that represent different aspects of participants' perceived barriers and facilitators to adherence. MK and the third author (JCF) agreed on a list of codes and then MK coded the material. All authors participated in condensing and summarizing the content of each code in order to make generalized descriptions concerning barriers and facilitators to TB treatment.

### Ethics

Informed consent was obtained from all participants prior to data collection. Anonymity and confidentiality were ensured. The study has been approved by the Regional Committees for Medical and Health Research Ethics (REK) in Norway and was granted ethical clearance by Addis Ababa Health Bureau in Ethiopia.

## Results

We found that interplay of factors is involved in the decision making about medication intake. Factors that influenced adherence to TB treatment positively were beliefs in the curability of TB, and in the severity of TB in the presence of HIV infection, support from families and health professionals. Barriers to treatment adherence were experiencing side effects, pill burden, economic constraints, stigma with lack of disclosure, and lack of adequate communication with health professionals. Table 1 presents a summary of study findings and their relationship to categories from our theoretical framework.

**Table 1:** Relationship between study findings and categories from theoretical framework

Theoretical framework	Related study findings
Accessibility of health care	Challenges of DOT

Evaluation of health care	Interaction with health personnel

Perception of symptoms and threat of disease	Beliefs about TB, TB treatment and co-infection

Social network characteristics	Social support, Stigma

Knowledge about disease	Beliefs about TB and co-infection

Demographic characteristics (social status, income and education)	Lack of food (poverty related)

Regimen factors	Pill burden, Side effects

Health care related factors	Interaction with health personnel, Challenges of DOT

### Beliefs about TB, TB treatment and co-infection

A majority of patients explained that TB could be cured, despite the presence of a concomitant HIV infection; but many also thought that it was more difficult to be cured when one had HIV. "Weakening" of the body by HIV was mentioned by one group of participants who explained that the two diseases "support each other" to weaken the body.

"If you have HIV, TB can be strong, because they can collide. If you do not have HIV, TB will disappear soon. Like any person who has TB, you take your drug, and it disappears, but if you have HIV, it will not disappear fast." (20 year-old female participant)

This in turn was mentioned by patients as being a reason to take TB medications properly when one was infected with HIV. A woman explained:

"You have to take what the doctors give you, you can not stop that. You can only live if you take the drugs. Two diseases, it will kill you at once, your body can not support that. We are different from those who have TB only." (50 year-old female participant)

Health professionals underlined the presence of this belief among patients, and said that many patients co-infected with TB and HIV are motivated to take their TB treatment properly because of the fear of getting severe TB symptoms because of the HIV.

There were divergent views among patients about which disease they thought was the most severe when comparing TB and HIV. One group mentioned HIV as being the most serious illness that they feared more, while another group thought that TB was more dangerous. Patients' views about severity affected the decision about which drugs to discontinue for those patients who decided to take one of the drugs and stop the other. A 36 years old male defaulter explained the reason why he decided to discontinue his anti-TB drugs and continue with the ART after experiencing side effects aggravated by lack of food:

"I have to take at least one of the drugs. I wanted to survive. I said, I will take the HIV drugs only. Interviewer: Why the HIV drugs and not the TB drugs? The HIV, it will kill you, it will kill you fast. Samba (TB) will not kill me if I take these drugs (ARV drugs) properly." (36 year-old male participant)

A factor that most health professionals believed helped patients to gain hope was the improvement they observed in other patients who had previously been critically ill. According to health professionals, that helped patients believe in the efficacy of treatment and hence gave them hope for themselves. A medical doctor stated:

"I think many patients are encouraged to come because they see critically ill patients with co-infection who have improved. That gives them a lot of hope." (medical doctor)

### Pill burden and side effects

Patients mentioned pill burden as being one of the major challenges of concomitant treatment, and they used expressions such as "becoming a drug bag", and "becoming a pharmacy". In addition to anti-TB drugs and ARV drugs, patients were also taking co-trimoxazole for PCP prophylaxis; and some in addition had to take drugs for other illnesses. From the perspective of some patients, a high number of pills was associated with potential damage to the body and a higher risk of not tolerating the drugs.

"Swallowing so many drugs, it was very difficult. I was scared that it would harm my body. Drugs can harm you if they are too many." (36-year-old male participant)

This belief was reinforced by some health professionals, as noted by a medical doctor:

"When they think about pill burden, they say, can I tolerate this, won't this kill me? Taking many pills is perceived as lethal by some. Patients worry a lot about whether their body will be able to handle so many pills every day." (medical doctor)

Forgetfulness and mix up of drugs as a result of high pill burden were also reported by some patients.

Side effects were experienced by more than half of the participants, mainly at the beginning of anti-TB treatment or upon initiation of concomitant treatment. Among side effects patients mentioned were generalized body weakness, burning of the stomach, "turning of the head", headache, bad dreams, rash and vomiting. A 57 year-old male participant who discontinued both his anti-TB and ART treatment after experiencing severe side effects explains:

"I continued the drugs but the nightmares and the hunger became too much. And then, I don't really even know how, my mind was also disturbed, so I took the drugs for some time, and stopped all drugs. You know the nightmare! And since I had no one beside me, and since the medications were disturbing me at night, and since it was pushing me to run away, I got scared and stopped the drugs." (57 year-old male participant)

### Lack of food

The majority of patients believed that lack of food or intake of inadequate food was associated with more severe side effects and a difficulty to tolerate the drugs. The amount and quality of food needed and the degree of possible side effects were also believed by some patients to be proportional to the drugs taken. Lack of food was mentioned as a factor adversely affecting their treatment by those patients who had insufficient income. Patients mentioned drugs could be harmful on an empty stomach, and that it was better not to take drugs if one had not eaten. Health professionals recognized the problem of food as being common, especially with the gain in appetite related to improvement. A clinical nurse explained:

"That (food) is a very serious problem for many of our patients. They almost want to kill us wanting help. They come here asking for help from NGOs. They say that especially TB drugs increase appetite. They feel hungry after they start feeling well a bit, and food becomes a problem." (nurse)

### Interaction with health personnel

The majority of patients in the study were happy about the way health professionals received and treated them at the health centers. Some said they were encouraged to come for treatment because health professionals were receiving them with a "good face", and were encouraging them to finish their treatment. A patient explained the support she received from health professionals:

"They [health professionals] were very good to me. They are like friends, did you not see? Since my head was not good, they were giving me the drugs in a certain way, in a bag, so that I know which drug to take when, they translated it in Amharic for me. They gave me a watch, they helped me a lot. They were like relatives. I have no words to thank them. I am standing today because of them. They told me what drugs to take at what time." (20 years old female patient)

When being asked about whether they received information about TB, or about the drugs and potential side effects, patients often replied that they did not get information. A patient said:

"Someone might be kind, that is not because they always give me drugs, they should also ask me what did it cause on you, is it good, did it cause any harm, what can we do for you? They have to say this. I come, they see my card, I get my drugs, and I go. That's all. Someone might be good naturally, but when I see from profession, it is less than I expected." (male focus group participant)

Few patients had asked health professionals at the TB clinics the information they needed. Those who asked questions tended to be more educated in general. For most of the other patients, the information that they had about TB and its treatment came from the community, from other patients at the health centers and mainly from the media. But many patients said that they were given information regarding ART and its side effects at the ART clinics. Health professionals said that adherence counseling is critical for patients, and that patients should be evaluated for their drug intake every time they come for a visit. Some nevertheless said that it had been difficult to do so, mainly because of time constraints. A clinical nurse said:

"What we do is not enough. The counseling should be done every time they come for treatment. Sometimes, we just give them their drug and send them home. You do not even know if they are taking the drugs the right way." (clinical nurse)

### The challenges of DOT

The majority of patients said that DOT with daily supervision of treatment for the first two months is a very difficult task to accomplish. The initial phase of treatment during which daily supervision is made was the phase when many patients were ill and weak, and for such patients, it meant that they often had to come to the clinic accompanied by their families. This meant that families were also bearing the burden of treatment. In addition to physical weakness, transport costs were also a problem for many patients who had financial difficulties:

"I had to come every day for injections. I tell you the truth, there was a time when I had to sell my jewellery: my rings, my necklace, everything. I had to run here without even eating breakfast." (35 year-old female patient)

DOT was particularly problematic for those patients who had irregular jobs and who had to go looking for a job on a daily basis. A 36 year-old daily laborer explained his situation:

"I have to go in the morning. We stand at the roundabout and wait for someone to give us a job. If I don't go, someone else will take it. It will not stand and wait for me." (36 year old male patient)

All health professionals agreed that DOT with daily supervision is a difficult task for most of their patients, and in particular co-infected patients who needed to attend the clinics for ART as well. Most believed that DOT is too rigid, and some stated that something needs to be done to modify the daily schedule into a better schedule for patients. A medical doctor explains:

"Asking a person to come every day is a punishment. We should change something; find a certain schedule, for example weekly for the first month." (medical doctor)

### Timing of ART

Participants in this study had started ART at different phases during TB treatment. Some were initiated on ART in the intensive phase of TB treatment, some in the continuation phase, and a few were already on ART when they were started on treatment for TB. Patients who had been told by health professionals that ART would be delayed until the end of the intensive phase of TB treatment were positive about this, since they had fears and doubts about starting concomitant treatment immediately. One group believed that their body would "adapt" to the TB drugs with time, and it will make it easier for them to tolerate the ART, hence allowing them to follow both treatments properly. Three of the patients who had defaulted treatment in this study said that initiation of ART in the intensive phase of their TB treatment had been too difficult, and that they were unable to tolerate treatment. They said that delaying the ART would have been better in their cases. A 36 years old male defaulter who had discontinued both anti-TB and ART drugs explained:

"It is better to take one drug and then the other drug. Your stomach can tolerate it. Your body can tolerate it. Together, it is too much. That is why I stopped my drugs." (36 year-old male participant)

### Stigma

Many patients believed that they were predisposed to stigma because of TB. For many of these patients, that was mainly due to the fact that people associated TB with HIV. Many had seen other HIV patients suffering from stigma and discrimination in their communities, and feared that the same might happen to them. Some shared their experiences, such as being pointed at in their neighborhoods, neighbors gossiping about their illness behind their backs and exclusion from social events. Some patients said the stigma was directly related to the TB because they were believed to be infectious. These actual experiences and anticipations of stigma resulted in many patients hiding their diagnosis of TB or only disclosing it to selected people, mostly close families. A male focus group participant said:

"Before, I knew a lot of people with TB who did not have HIV. But now, if you say you have TB, people will take it to HIV. Me, even when I take these TB drugs, I don't tell to anyone." (male focus group participant)

Fear of stigma not only lead to such patients not disclosing their illnesses but also made seeking TB treatment in their catchments areas difficult as mentioned earlier, for fear of being identified by neighbors:

"I don't want neighbors to see me here [at the TB clinic]. One day, I saw this girl from my neighborhood here; I was sitting outside and waiting. I wished the earth would open and swallow me. I know she would spread the gossip. I tried to hide behind the man sitting next to me. I don't think she saw me." (36 years old male patient)

This fear was confirmed by many health professionals who explained that they have faced difficulties with patients who do not want to receive treatment in the health centers located in their vicinity, for fear of being identified by neighbors.

### Social support

Social support was found to be crucial for patients' treatment. Some patients had been seriously ill when treatment was initiated and needed someone to accompany them for treatment. In addition, families provided food and transportation money since some patients had no income and others had to stop working for some time. Families were also a source of encouragement and comfort for those patients who had lost hope.

"My family members supported me a lot. They encouraged me. After the TB, when they found the HIV, I wanted to die. I did not want to live. But my families, especially my brothers and sisters, they said: you are not the only one. Look, many people have this. It is because of them that I am alive today." (female focus group participant)

Some health professionals also noted that patients who had family support and come to the clinics accompanied are usually those who successfully complete their treatment.

## Discussion

Our findings suggest that an interplay of factors is involved in patients' decision- making about use of concomitant treatment for TB and HIV. For the individual patient, barriers act against a set of facilitating factors, and the final decision about treatment depends on which factors predominate. Although some of the factors are related to poverty, and are more difficult to tackle, other factors are liable to improvement through intervention. Factors associated with adherence we have identified in this study have previously been described in the literature on adherence to TB treatment in different contexts. These include side effects, rigidity of DOT, social support and interaction with health personnel [12,16-23]. Other factors appear to be more specific for patients who use concomitant treatment for TB and HIV, such as beliefs about concomitant illness and the experience of stigma. Our focus in this study was adherence to TB treatment in co-infected patients on concomitant treatment for TB and HIV. Adherence to treatment of one illness was found to affect adherence to the treatment of the other illness: among the defaulters, one patient was found who gave up TB treatment and continued ART after weighing in benefits and costs associated with disease severity; and other patients had decided to discontinue both treatments.

### TB as a curable condition

Patients' belief that TB was a curable illness appeared to be a motivating factor for adherence. Although some authors had raised concern about the possibility of the relation between TB and HIV enhancing the questioning of the curability of TB [29], patients did not convey such views in our study. Studies have shown that belief in the efficacy of treatment facilitates adherence to TB treatment [11,27,30]; while other studies describe how patients' adherence was adversely affected by belief that TB is incurable or the treatment inefficient, or that alternative treatment such as traditional medicine is better [11,27,29-31]. Our findings suggest that informing patients explicitly that TB is a curable disease might motivate them for treatment.

### Adherence counseling and information

Pill burden and side effects were major challenges to concomitant treatment. The adverse impacts of pill burden on adherence to treatment have previously been documented [8,15,32,33] and so have those of both perceived and experienced side effects [11,18,34-38]. The situation is further aggravated if health professionals do not warn patients about side-effects. Our findings suggest that adherence counseling might facilitate adherence. TB and HIV clinics should work in collaboration to provide patients with uniform and complete information. Patients should be well informed about co-infection and concomitant treatment. Side effects, pill burden and timing of ART in the course of TB treatment should be thoroughly addressed. Beliefs related to amount and quality of food that needs to be consumed should be addressed. The importance of social support for adherence to treatment should also be discussed with patients.

### Timing of ART

The national recommendation for the initiation of ART in patients on anti-TB is to start patients with CD4 of <200 on ART as soon as TB treatment is tolerated (usually between 2 and 8 weeks); for those with a CD4 count between 200 and350, ART is to be initiated once the intensive phase of anti-TB is completed. ART is deferred for those with CD4 greater than 350.Those already on ART would continue their treatment [39].

We found that improper timing of ART treatment after initiation of TB treatment could be a potential barrier to patients' adherence. Health personnel should discuss the timing of ART with the individual patient, in order to ensure adherence to both treatments. Experts' concern about adherence when multiple medications are started at the same time has been used in determining timing of ART during TB treatment in some guidelines [5], and not actual evidences from studies, and this needs to be further explored. In addition, patients started on ART drugs early in their TB treatment can also be predisposed to Immune Reconstitution Inflammatory Syndrome, presenting as transient worsening or appearance of new signs and symptoms of TB [40]; this and other clinical risks such as risk of mortality thus need to be taken into consideration when deciding the timing of ART treatment.

### Stigma

Stigma is a potential barrier to treatment because it makes patients reluctant to attend treatment in clinics located in their neighborhoods and leads to non-disclosure of illness. Disclosure can play a positive role because it can help the patient mobilize social support thereby facilitating adherence; and is also important for public health reasons such as avoidance of further infection transmission. But health professionals should be aware of the negative impacts of self-disclosure in social environments where disease related stigma is widespread. In a situation where consequences of disclosure are uncertain, it may be rational for a patient not to disclose or to have very selective disclosure. Studies have highlighted the negative impacts of HIV-related stigma on TB treatment in co-infected patients. A study from Thailand showed that although both TB and HIV were stigmatized, more stigma was attached to HIV, and that patients got delayed in seeking treatment or defaulted from treatment of TB when they suspected they have HIV and feared for it to be detected [41]. A study from Nigeria showed that one of the reasons for default in TB patients with HIV was relocation secondary to HIV related stigma and discrimination [42]. There is a need to address both TB and HIV related stigma in the local context, including through information and communication using different channels.

### The burden of treatment

DOT was found to be particularly challenging for this group of patients, because of physical demands and economic constraints; and because further attendances at the clinic for ART were required. The literature had previously documented the adverse impacts of DOT on adherence to treatment [21,23]. A review of trials comparing DOT with self supervision of treatment showed that there was no difference in treatment completion between the two groups, as well as between patients supervised by different groups of individuals (health professional, lay health worker, family/community member) [43]. Looking for alternative sources of supervision such as community based supervision is necessary and encouraging results have already been obtained in a community based DOTS program in the Oromia Region in Ethiopia [2].

### Food

Our results suggest that providing early support in terms of food and financial assistance to at least cover transportation costs for patients with financial difficulties might facilitate treatment. A number of NGOs are already in place providing such supports for HIV patients and their efforts should be encouraged and strengthened. Health workers in the treatment facilities could recommend to these NGOs that HIV patients receiving concomitant TB and HIV treatment be given special attention.

### Methodological considerations

This study is based on interviews with a total of 29 patients and nine health professionals, and is limited to one geographical area. But we think that our results reflect diversity in views and experiences; we also triangulated data sources in order to strengthen the validity of our results. We thus believe our study can provide valuable insights into the experiences of TB medication intake among co-infected patients on concomitant treatment in other settings. In addition, there is a need to conduct quantitative studies to further explore the matter and obtain generalizable results; and we believe the findings of this study will be of paramount importance for such studies.

## Conclusion

Health professionals and policy makers should be aware of factors influencing TB treatment in TB/HIV co-infected patients on concomitant treatment for TB and HIV. Our findings suggest that provision of food and minimal financial support might facilitate adherence. Counseling might also facilitate adherence, in particular for those who start ART in the early phases of TB treatment, and beliefs related to side-effects and pill burden should be addressed. Information to the public may reduce TB and HIV related stigma.

Conducting intervention studies addressing these different dimensions and documenting the impacts on adherence is recommended.

## Competing interests

The authors declare that they have no competing interests.

## Authors' contributions

MK designed the study in collaboration with GAB and JCF. MK collected the data. MK coded the data and all authors contributed in the subsequent analysis. All authors have critically revised the manuscript.

## Pre-publication history

The pre-publication history for this paper can be accessed here:

http://www.biomedcentral.com/1471-2458/10/651/prepub

## References

[B1] World Health Organization Global Tuberculosis Control: surveillance, planning, financing 2008 Geneva: World Health Organization

[B2] Federal Ministry of Health of Ethiopia Manual for tuberculosis, leprosy and TB/HIV prevention and control program 2008 Addis Ababa: Federal Ministry of Health

[B3] Corbett EL Marston B Churchyard GJ De Cock KM Tuberculosis in Sub-Saharan Africa: opportunities, challenges and changes in the era of antiretroviral treatment Lancet 2006 367 926 937 10.1016/S0140-6736(06)68383-9 16546541

[B4] Dean GL Edwards SG Ives NJ Matthews G Fox EF Navaratne L Fisher M Taylor GP Miller R Taylor CB de Ruiter A Pozniak AL Treatment of tuberculosis in HIV infected persons in the era of highly active antiretroviral therapy AIDS 2002 16 75 83 10.1097/00002030-200201040-00010 11741165

[B5] Kwara A Flanigan TP Carter EJ Highly active antiretroviral therapy (HAART) in adults with tuberculosis: current status Int J Tuberc Lung Dis 2005 9 248 257 15786886

[B6] Breen RA Miller RF Gorsuch T Smith CJ Schwenk A Holmes W Ballinger J Swaden L Johnson MA Cropley I Lipman MC Adverse events and treatment interruption in tuberculosis patients with and without HIV co-infection Thorax 2006 6 791 794 10.1136/thx.2006.058867 PMC2117099 16844730

[B7] Aaron L Saadoun D Calatroni I Launay O Mémain N Marchal G Dupont B Bouchaud O Valeyre D Lortholary O Tuberculosis in HIV infected patients: a comprehensive review Clin Microbiol Infect 2004 10 388 398 10.1111/j.1469-0691.2004.00758.x 15113314

[B8] Ingersoll KS Cohen J The impact of medication regimen factors on adherence to chronic treatment: a review of literature J Behav Med 2008 31 213 224 10.1007/s10865-007-9147-y 18202907 PMC2868342

[B9] Claxton AJ Cramer J Pierce C A systematic review of the associations between dose regimens and medication compliance Clin Ther 2001 23 1296 1310 10.1016/S0149-2918(01)80109-0 11558866

[B10] Richter A Anton SE Koch P Dennett SL The impact of reducing dose frequency on health outcomes Clin Ther 2003 25 2307 2335 10.1016/S0149-2918(03)80222-9 14512137

[B11] Munro SA Lewin SA Smith HJ Engel ME Fretheim A Volmink J Patient adherence to tuberculosis treatment: a systematic review of qualitative research PLoS Med 2007 4 e238 10.1371/journal.pmed.0040238 17676945 PMC1925126

[B12] Shargie EB Lindtjørn B Determinants of treatment adherence among smear positive pulmonary tuberculosis patients in Southern Ethiopia PLoS Med 2007 4 e37 10.1371/journal.pmed.0040037 17298164 PMC1796905

[B13] World Health Organization Adherence to Long Term Therapies: Evidence for Action 2003 Geneva: World Health Organization http://whqlibdoc.who.int/publications/2003/9241545992.pdf

[B14] Farmer P Social scientists and the new tuberculosis Soc Sci Med 1997 44 347 358 10.1016/S0277-9536(96)00143-8 9004369

[B15] Chesney MA Morin M Sherr L Adherence to HIV combination therapy Soc Sci Med 2000 50 1599 1605 10.1016/S0277-9536(99)00468-2 10795966

[B16] Demissie M Kebede D Defaulting from tuberculosis treatment at the Addis Abeba Tuberculosis Centre and factors associated with it Ethiop Med J 1994 32 97 106 8033883

[B17] Getahun H Aragaw D Tuberculosis in rural northwest Ethiopia: community perspective Ethiop Med J 2001 39 283 291 12380228

[B18] Tekle B Mariam DH Ali A Defaulting from DOTS and its determinants in three districts of Arsi Zone in Ethiopia Int J Tuberc Lung Dis 2002 6 573 579 12102295

[B19] Shargie EB Lindtjørn B Determinants of treatment adherence among smear positive pulmonary tuberculosis patients in Southern Ethiopia PLoS Med 2007 4 e37 10.1371/journal.pmed.0040037 17298164 PMC1796905

[B20] Michael KW Belachew T Jira C Tuberculosis defaulters from the "dots" regimen in Jimma zone, southwest Ethiopia Ethiop Med J 2004 42 247 253 16124124

[B21] Getahun H Maher D Contribution of "TB clubs" to tuberculosis control in a rural district in Ethiopia Int J Tuberc Lung Dis 2000 4 174 178 10694097

[B22] Demissie M Getahun H Lindtjorn B Community tuberculosis care through "TB clubs" in rural north Ethiopia Soc Sci Med 2003 56 2009 2018 10.1016/S0277-9536(02)00182-X 12697193

[B23] Sagbakken M Frich JC Bjune G Barriers and enablers in the management of tuberculosis treatment in Addis-Ababa, Ethiopia: a qualitative study BMC Public Health 2008 8 11 10.1186/1471-2458-8-11 18186946 PMC2257959

[B24] Addis Ababa Health Bureau Strategic plan of Addis-Ababa City Administration Health Bureau 2004-2007 2007 Addis Ababa: Addis Ababa Health Bureau

[B25] Federal Ministry of Health, Federal HIV/AIDS Prevention and Control Office Guidelines For implementation of the antiretroviral therapy programme in Ethiopia 2007 Addis Ababa: Federal Ministry of Health

[B26] Cummings KM Becker MH Maile MC Bringing the models together: an empirical approach to combining variables used to explain health actions J Behav Med 1980 3 123 145 10.1007/BF00844986 7420418

[B27] Noyes J Popay J Directly observed therapy and tuberculosis: how can a systematic review of qualitative research contribute to improving services? J Adv Nurs 2007 57 227 243 10.1111/j.1365-2648.2006.04092.x 17233644

[B28] Malterud K Shared understanding of the qualitative research process. Guidelines for the medical researcher Fam Pract 1993 10 201 206 10.1093/fampra/10.2.201 8359612

[B29] Liefooghe R Baliddawa JB Kipruto EM Vermeire C De Munynck AO From their own perspective. A Kenyan community's perception of tuberculosis Trop Med Int Health 1997 2 809 821 10.1046/j.1365-3156.1997.d01-380.x 9294551

[B30] Rowe KA Makhubele B Hargreaves JR Porter JD Hausler HP Pronyk PM Adherence to TB preventive therapy for HIV-positive patients in rural South Africa: implications for antiretroviral delivery in resource-poor settings? Int J Tuberc Lung Dis 2005 9 263 269 15786888

[B31] Edginton ME Sekatane CS Goldstein SJ Patients' beliefs: do they affect tuberculosis control? A study in a rural district of South Africa Int J Tuberc Lung Dis 2002 6 1075 1082 12546115

[B32] Mehta S Moore RD Graham NM Potential factors affecting adherence with HIV therapy AIDS 1997 11 1665 1670 10.1097/00002030-199714000-00002 9386800

[B33] Chesney M Adherence to HAART regimens AIDS Patient Care STDS 2003 17 169 177 10.1089/108729103321619773 12737640

[B34] Rocha M Pereira S Ferreira L Barros H The role of adherence in tuberculosis HIV positive patients treated in ambulatory regimen Eur Respir J 2003 21 785 788 10.1183/09031936.03.00077302 12765421

[B35] Ngamvithayapong J Uthaivoravit W Yanai H Akarasewi P Sawanpanyalert P Adherence to tuberculosis preventive therapy among HIV-infected persons in Chiang Rai Thailand AIDS 1997 11 107 112 10.1097/00002030-199701000-00016 9110083

[B36] Wares DF Singh S Acharya AK Dangi R Non-adherence to tuberculosis treatment in the eastern Tarai of Nepal Int J Tuberc Lung Dis 2003 7 327 335 12729337

[B37] Kaona FA Tuba M Siziva S Sikaona L An assessment of factors contributing to treatment adherence and knowledge of TB transmission among patients on TB treatment BMC Public Health 2004 4 68 10.1186/1471-2458-4-68 15625004 PMC545081

[B38] Hardon AP Akurut D Comoro C Ekezie C Irunde HF Gerrits T Kglatwane J Kinsman J Kwasa R Maridadi J Moroka TM Moyo S Nakiyemba A Nsimba S Ogenyi R Oyabba T Temu F Laing R Hunger, waiting time and transport costs: time to confront challenges to ART adherence in Africa AIDS Care 2007 19 658 665 10.1080/09540120701244943 17505927

[B39] Federal Ministry of Health, Federal HIV/AIDS Prevention and Control Office Guidelines for Management of Opportunistic Infections and Antiretroviral Treatment in Adolescents and Adults in Ethiopia 2007 Federal Ministry of Health

[B40] Corbett EL Marston B Churchyard GJ De Cock KM Tuberculosis in sub-Saharan Africa: opportunities, challenges, and change in the era of antiretroviral treatment Lancet 2006 367 926 937 10.1016/S0140-6736(06)68383-9 16546541

[B41] Ngamvithayapong J Winkvist A Diwan V High AIDS awareness may cause tuberculosis patient delay: results from an HIV epidemic area, Thailand AIDS 2000 14 1413 1419 10.1097/00002030-200007070-00015 10930157

[B42] Daniel OJ Alausa OK Treatment outcome of TB/HIV positive and TB/HIV negative patients on directly observed treatment, short course (DOTS) in Sagamu, Nigeria Niger J Med 2006 15 222 226 17111747 10.4314/njm.v15i3.37217

[B43] Volmink J Garner P Directly observed therapy for treating tuberculosis Cochrane Database Syst Rev 2007 CD003343 17943789 10.1002/14651858.CD003343.pub3

